# BRAF^V600E^ mutation analysis in fine‐needle aspiration cytology specimens for diagnosis of thyroid nodules: The influence of false‐positive and false‐negative results

**DOI:** 10.1002/cam4.2478

**Published:** 2019-08-08

**Authors:** Chong‐Ke Zhao, Jia‐Yi Zheng, Li‐Ping Sun, Rong‐Ying Xu, Qing Wei, Hui‐Xiong Xu

**Affiliations:** ^1^ Department of Medical Ultrasound, Shanghai Tenth People's Hospital, Ultrasound Research and Education Institute Tongji University School of Medicine Shanghai China; ^2^ Thyroid Institute Tongji University School of Medicine Shanghai China; ^3^ Shanghai Center for Thyroid Diseases Shanghai China; ^4^ Department of Pathology, Shanghai Tenth People's Hospital Tongji University School of Medicine Shanghai China

**Keywords:** BRAF^V600E^ mutation, diagnosis, fine‐needle aspiration cytology, polymerase chain reaction, thyroid nodule

## Abstract

**Background:**

The accurate evaluation of BRAF^V600E^ mutation in preoperative fine‐needle aspiration cytology (FNAC) specimens is important for making management decisions in thyroid nodules (TNs). The aim of this study was to assess the false‐positive and false‐negative BRAF^V600E^ mutations in thyroid FNAC specimens and their influence on diagnosis of TN.

**Methods:**

This prospective study enrolled 292 nodules in 269 patients who underwent BRAF^V600E^ mutation analysis using amplification refractory mutation system‐quantitative real‐time polymerase chain reaction (ARMS‐qPCR) both in FNAC specimens and formalin‐fixed, paraffin‐embedded (FFPE) tissue samples after surgery. The false‐positive and false‐negative mutations for BRAF^V600E^ analysis using ARMS‐qPCR in FNAC specimens were recorded, with reference to the results of BRAF^V600E^ mutation analysis using ARMS‐qPCR in FFPE tissue sample. Diagnostic performances of FNAC, BRAF^V600E^ mutation analysis in FNAC specimens, BRAF^V600E^ mutation analysis in FFPE tissue sample, and the combination of FNAC and BRAF^V600E^ mutation analysis for predicting thyroid malignancy were assessed.

**Results:**

The false‐positive and false‐negative mutations for BRAF^V600E^ analysis using ARMS‐qPCR in FNAC specimens were 10.1% (19/189) and 7.1% (7/98), respectively. FNAC combined with preoperative BRAF^V600E^ mutation analysis significantly increased the diagnostic sensitivity from 75.7% to 92.3%, and accuracy from 78.7% to 90.6% in comparison with FNAC alone (both *P* < .001). No significant differences were found between the combination of FNAC and BRAF^V600E^ mutation analysis in FNAC specimens and the combination of FNAC and BRAF^V600E^ mutation analysis in FFPE tissue sample (sensitivity: 92.3% vs 91.9%; accuracy: 90.6% vs 91.3%; both *P* > .05).

**Conclusions:**

FNAC combined with preoperative BRAF^V600E^ mutation analysis can significantly increase the diagnostic performance in comparison with FNAC alone. False‐positive and false‐negative BRAF^V600E^ mutation results are found in preoperative FNAC specimens, whereas it does not affect the overall auxiliary diagnosis of TNs.

## Graphical abstract

The false‐positive and false‐negative mutations for BRAFV600E analysis in thyroid (FNAC) specimens using amplification refractory mutation system‐quantitative real‐time polymerase chain reaction (ARMS‐qPCR) were 10.1% (19/189) and 7.1% (7/98), respectively. The false‐positive and false‐negative BRAFV600E mutation results in FNAC specimens using ARMS‐qPCR do not affect the overall auxiliary diagnosis of thyroid nodules (TNs).

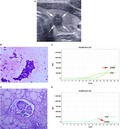

## 1 INTRODUCTION

Ultrasound (US)‐guided fine‐needle aspiration cytology (FNAC) is a standard diagnostic method for thyroid nodules (TNs), but some cytological results such as nondiagnostic, indeterminate, and false‐negative results might cause confusion in the management of TNs.^1^, ^2^ To overcome these limitations, several molecular markers have been applied in combination with FNAC results.^3^ Among various molecular markers related to thyroid cancer, BRAF^V600E^ mutation is the most common molecular marker found in papillary thyroid carcinoma (PTC).^1^, ^4^ Because the BRAF^V600E^ test has a high positive predictive value (PPV), it can be used to improve the diagnostic accuracy of FNAC and to overcome the limitations of FNAC as mentioned above.^3^, ^5^, ^6^ In addition, many studies have found that the presence of a BRAF^V600E^ mutation is associated with more aggressive tumor characteristics, such as extrathyroidal extension, lymph node involvement,^7^ resistance to radioactive iodide,^8^ PTC recurrence, 9 and PTC‐related mortality.^10^ Some authors advocated preoperative BRAF^V600E^ mutation analysis as an aid for improving risk stratification of PTC patients in order to define a more individualized treatment plan.^11^, ^12^, ^13^ Therefore, it is very important to evaluate the accuracy of BRAF^V600E^ mutation analysis in preoperative FNAC specimens.

Conventional Sanger sequencing is a standard method to detect the BRAF^V600E^ mutation because of its high reliability.^14^ However, this molecular technique is not sensitive enough for the detection of low frequency mutations (less than 20%) in the sample, which leads to a higher false‐negative result.^15^ Moreover, this methodology is not suitable for batch testing, which limits its clinical application.^16^ Of note, the BRAF^V600E^ mutation is present only in the malignant cells in thyroid FNAC specimens which are admixed with other cell types and blood. Therefore, a highly sensitive assay is required to detect the BRAF^V600E^ mutations in FNAC specimens. Efforts to increase sensitivity have produced methods that can detect BRAF^V600E^ with a limit of detection (LOD) in as few as 0.1%‐2% of the total cell population. However, false‐positive mutations for the BRAF^V600E^ mutation are present in 0.08%‐5.4% of the cases when highly sensitive analytic methods such as pyrosequencing,^12^ polymerase chain reaction‐restriction fragment length polymorphism (PCR‐RFLP),^17^ dual priming oligonucleotide (DPO)‐based multiplex PCR,^5^, ^6^, ^14^ mutant enrichment with 3'‐modified oligonucleotide (MEMO) sequencing,^6^ real‐time PCR,^18^ or droplet digital PCR (ddPCR) 19 are used.

Recently, the amplification refractory mutation system‐quantitative real‐time PCR (ARMS‐qPCR) method is developed to specifically detect the BRAF^V600E^ mutation with high sensitivity (0.1%‐1% of total cells), which can provide quantitative and rapid (<4 hour) result. And, it has been commercially used at a low cost of $87/test in clinical practice.^13^, ^20^, ^21^, ^22^ However, there has been no report evaluating the false‐positive and false‐negative mutation results of BRAF^V600E^ analysis in thyroid FNAC specimens before surgery using ARMS‐qPCR until now, which may cause uncertainty in interpreting the BRAF^V600E^ analysis and the following management. Thus, the purpose of this study was to assess the false‐positive and false‐negative BRAF^V600E^ mutation results using ARMS‐qPCR in preoperative thyroid FNAC specimens, with reference to the BRAF^V600E^ mutation results using ARMS‐qPCR in formalin‐fixed, paraffin‐embedded (FFPE) tissue samples after surgery. In addition, we also investigated whether those false results would affect the added diagnostic value of BRAF^V600E^ mutation analysis to FNAC for evaluation of TNs.

## 2 MATERIALS AND METHODS

### 2.1 Patients

This prospective study was approved by the Ethics Committee of the University Hospital and written informed consent was obtained from all the patients before US‐guided FNAC and genetic analysis.

**Figure 1:**
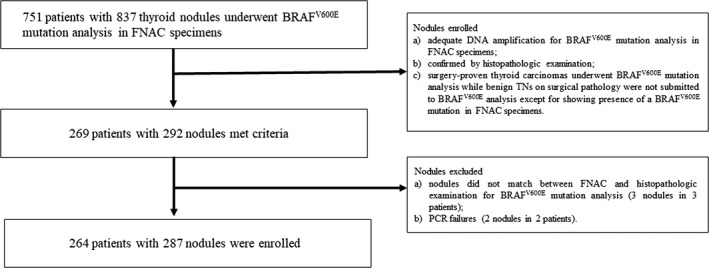
Flowchart of patient enrollment and exclusion

From December 2016 to December 2017, 751 patients with 837 nodules had undergone both US‐guided FNAC and preoperative BRAF^V600E^ mutation analysis. The inclusion criteria were as follows: (a) adequate DNA amplification for BRAF^V600E^ mutation analysis in FNAC specimens; (b) histopathologic examination and diagnosis of the TN after surgery; (c) surgery‐proven thyroid carcinomas underwent BRAF^V600E^ mutation analysis while benign TNs on surgical pathology were not submitted to BRAF^V600E^ analysis except for showing the presence of a BRAF^V600E^ mutation in FNAC specimens. BRAF^V600E^ mutational analysis was performed using ARMS‐qPCR both in the FNAC specimens and FFPE tissue samples. In total, 269 patients with 292 nodules were enrolled. Three nodules in three patients were excluded because the nodules subject to FNAC for BRAF^V600E^ mutation analysis did not match those subject to histopathologic examination for BRAF^V600E^ mutation analysis. Additionally, two nodules in two patients were excluded because of PCR failures. Finally, the study group consisted of 264 patients with 287 TNs (Figure 1). Of the 264 patients, one nodule per patient was analyzed in 241 patients, and two nodules per patient were analyzed in 23 patients.

### 2.2 US‐guided FNAC procedure

All FNAC procedures were performed under US guidance by one of the three radiologists who had more than 3 years experience in thyroid FNAC and more than 5 years experience in thyroid US. Using a high‐frequency linear array transducer with the US instrument, the FNAC specimens were obtained from the nodule with a 5‐mL syringe. Local anesthesia was performed routinely with 1% lidocaine. The needle was inserted into the nodule and moved rapidly back and forth in different directions within the nodule when suction was applied. For BRAF^V600E^ mutation analysis, after acquisition of the optimal amount for cytological diagnosis, additional aspiration was performed to isolate genomic DNA. The FNAC aspirates were expressed onto frosted‐end glass slides and immediately fixed in 95% alcohol for hematoxylin and eosin (H&E) staining.

One of the three cytopathologists experienced in thyroid cytology reviewed all the FNAC specimens in the Department of Pathology of the University Hospital. According to the Bethesda System for Reporting Thyroid Cytopathology (TBSRTC) criterion, cytological diagnoses consist of six categories 23: (I) Nondiagnostic; (II) Benign; (III) Atypia of undetermined significance/follicular lesion of undetermined significance (AUS/FLUS); (IV) Follicular neoplasm or suspicious for follicular neoplasm (FN/SFN); (V) Suspicious for malignancy (SUSP); (VI) Malignant. The criterion for an adequate smear was the presence of six groups of cells with more than 10 cells per group.

### 2.3 Detection of BRAF^V600E^ mutation by ARMS‐qPCR in FNAC specimens

BRAF^V600E^ mutation analysis was also performed at the Department of Pathology in the University Hospital by a single PCR operator who had more than 3 years experience in BRAF^V600E^ mutation analysis of thyroid specimens.

#### 2.3.1 Manual macrodissection of the FNAC slide

The “manual macrodissection” methodology chosen for the molecular study has been previously described and is routinely used in the laboratory.^24^ Firstly, the tumor cells or suspicious cells from H&E staining FNAC smears were identified and selected by cytopathologists using microscopy to assure adequate thyroid cell representation. After choosing the most representative slide or marking the regions of the slides containing numerous lesional thyrocytes, the slides were deparaffinized using xylene and ethanol. Once the slide was air dried, lysis solution without proteinase K was poured on the slide to scrape material from the entire smear or marked area of the slide using a single‐edged razor blade. Finally, the scraped tissues were collected to a microcentrifuge tube including lysis solution with proteinase K for the DNA extraction and analysis.

#### 2.3.2 DNA extraction, quantification, and dilution

DNA extraction was successfully completed in all samples following the manufacturer's instructions with a DNA extraction kit (ADx‐FF01). After DNA isolation, its concentration was assessed using a NanoDrop 2000 spectrophotometer (Thermo). For identification of DNA purity and quality, the spectral absorbance (OD value) and the 260/280 and 260/230 ratios were calculated. The purity of DNA was considered high if the OD 260/280 ratio was close to 1.8, whereas an OD 260/280 ratio of >2.0 is suggestive for RNA contamination, and an OD 260/280 ratio of <1.6 is indicative of organic solvent contamination. DNA with an OD 260/230 >2.0 was diluted to ~1 ng/μL with elution buffer ATE (QIAGEN). The extracted DNA was stored in a −20°C refrigerator (Haier Pharmaceutical Co., Ltd.) until used.

#### 2.3.3 BRAF^V600E^ mutation detection

A validated, China Food and Drug Administration (CFDA)‐approved fluorescent PCR detection Kit (ADx‐ARMS, Amoy Diagnostics Co. Ltd) based on ARMS technique was used for the detection of the BRAF^V600E^ mutation in the samples. The ARMS‐qPCR is a highly sensitive method employed for specifically detecting the BRAF^V600E^ (c.T1799A in exon 15). Using specific primers, the ARMS‐qPCR amplifies the target sequence and the amplified products are analyzed by amplicon‐specific fluorescent probes (double loop probes). Briefly, each PCR reaction system included 5 μL of extracted DNA, 0.4‐μL TaqDNA polymerase, and 35‐μL reaction mixture in a kit containing oligonucleotide primers, dNTPs, double loop probes, MgCl_2_, ammonium sulfate, and potassium chloride. The PCR reaction was carried out on ABI 7900 Fast real‐time fluorescence‐PCR machine (Applied Biosystems) with the following conditions: cycle 1 with an initial denaturation step at 95°C for 5 minutes; cycle 2:15 annealing cycles at 95°C for 25 seconds, 64°C for 20 seconds, and 72°C for 20 seconds, followed by cycle 3:31 extension cycles at 93°C for 25 seconds, 60°C for 35 seconds, 72°C for 20 seconds. The FAM (mutation type) and VIC (wild‐type) signals were captured at 60°C during cycle 3. Fluorescence increases geometrically corresponding to the exponential increase of the PCR products, which is used to determine the threshold cycle (Ct value). The Ct value was calculated automatically by this system at the end of the reaction. Each run contained a negative control and a positive control.

If the collected signals met the following three criteria, the testing was regarded as success and the results trustworthy: Firstly, the VIC signal and FAM signal of the positive control rose and the Ct value of the FAM signal was less than 20. Secondly, the VIC signal and FAM signal of the negative control did not rise. Thirdly, the VIC signals of the sample and the positive control were supposed to rise and the VIC signal Ct value of the sample ranged from 13 to 21. If the Ct value of the FAM signal in the sample was less than 28, it was considered to indicate the presence of a BRAF^V600E^ mutation, otherwise the sample was deemed to be negative for BRAF^V600E^.

### 2.4 Detection of BRAF ^V600E^ mutation by ARMS‐qPCR in FFPE tissue samples

The ARMS‐qPCR analysis for BRAF^V600E^ mutation was performed on the same nodule as the preoperative FNAC by macrodissection on FFPE slides after surgery. Four sections (5‐μm thick) were cut from FFPE tumor tissue blocks and were mounted onto microscopic slides. One section slide was stained with H&E for histopathological examination. The H&E staining section was used as a reference, and tumor‐rich regions of the sections were chosen from the three slides based on H&E staining patterns. Three 5‐μm thick FFPE slides were deparaffinized using xylene and ethanol. The tumor‐rich regions of the three sections slides were scraped using a single‐edged razor blade and were collected into a microcentrifuge tube. DNA extraction, quantification and dilution, and BRAF^V600E^ mutation detection procedure in FFPE tissue samples were similar as those in FNAC specimens except for DNA was diluted to ~2 ng/μL with elution buffer ATE (QIAGEN).

### 2.5 Statistical analysis

BRAF^V600E^ mutation analysis in the histological diagnosis after surgery was used as the reference standard. The following four entities were considered: True‐positive mutation = BRAF^V600E^ mutations detected both in FNAC specimens and FFPE tissue samples; True‐negative mutation = no BRAF^V600E^ mutations detected both in FNAC specimens and FFPE tissue samples; False‐positive mutation = BRAF^V600E^ mutations detected in FNAC specimens but not in FFPE tissue samples; False‐negative mutation = BRAF^V600E^ mutations negative in FNAC specimens, whereas positive in FFPE tissue samples. The rates of true‐positive mutation, true‐negative mutation, false‐positive mutation, and false‐negative mutation were calculated.

Statistical analyses were performed with SPSS version 19.0 software (IBM Corporation, Armonk, NY). With the histopathological analysis after surgery as the reference standard, the sensitivity, specificity, PPV, negative predictive value (NPV), and accuracy were calculated by the diagnostic test 2 × 2 contingency table. With regard to FNAC results for predicting thyroid malignancy, FNAC results of SUSP and malignant cytology were considered as malignant, whereas the others were considered as benign. With regard to BRAF^V600E^ analysis in FNAC specimens or FFPE tissue samples for predicting thyroid malignancy, BRAF^V600E^‐positive mutation was considered as malignancy, whereas BRAF^V600E^‐negative mutation was considered as benign. For the combination of FNAC results and BRAF^V600E^ analysis in FNAC specimens or FFPE tissue samples for predicting thyroid malignancy , either of two methods diagnosed the nodule as malignant was regarded as malignant, while only both two methods diagnosed the nodule as benign was regarded as benign. The McNemar test was used to compare the differences in sensitivity, specificity, and accuracy, whereas Chi‐squared test or Fisher exact test was used in PPV and NPV. A *P*‐value of .05 or less was considered to indicate a statistically significant difference.

## 3 RESULTS

### 3.1 Final diagnosis

The 264 patients included 62 men and 202 women with a mean age ± standard deviation of 46.4 ± 13.6 years (range, 19‐75 years). The mean size of nodules at the longest diameter was 10.8 ± 6.7 mm (range, 5‐48 mm) (Table 1).

**Table 1:** Basic characteristics of patients and nodules

Characteristic	
Patients, n	264
Patient sex	
Men, n	62
Women, n	202
Age, yr	46.4 ± 13.6 (19‐75)
Nodules, n	287
Nodule size, mm	10.8 ± 6.7 (5‐48)
Malignant nodules, n	222
Papillary thyroid Carcinoma, n (%)	218 (98.2)
Follicular thyroid carcinoma, n (%)	3 (1.4)
Anaplastic thyroid carcinoma, n (%)	1 (0.4)
Benign nodules, n	65
Nodular hyperplasia, n (%)	32 (49.2)
Hashimoto's nodule, n (%)	14 (21.6)
Adenomatous hyperplasia, n (%)	13 (20)
Follicular adenoma, n (%)	4 (6.2)
Subacute thyroiditis nodule, n (%)	1 (1.5)
Parathyroid nodule, n (%)	1 (1.5)

Note

Data are presented as mean ± SD (range) where applicable.

Abbreviation: n, number.

Sixty‐five nodules were histologically confirmed as benign, and 222 nodules confirmed by pathological specimens were malignant. The benign nodules included 32 nodular hyperplasias, 14 Hashimoto's nodules, 13 adenomatous hyperplasias, four follicular adenomas, one subacute thyroiditis nodule, and one parathyroid nodule. Thyroid malignancy consisted of 218 PTCs, three follicular thyroid carcinomas (FTCs), and one anaplastic thyroid carcinoma (ATC) (Table 1).

### 3.2 FNAC results

Of 287 FNAC analyses performed (Table 2), 12 (4.2%) nodules were reported to be nondiagnostic (Bethesda I), 38 (13.6%) nodules benign cytology (Bethesda II), 108 (37.6%) indeterminate cytologies (57 [19.9%] AUS/FLUS [Bethesda III], 5 [1.7%] FN/SFN [Bethesda IV], and 46 [16.0%] SUSP [Bethesda V]), and 129 (45.6%) malignant cytology (Bethesda VI).

**Table 2:** The results of preoperative BRAF^V600E^ mutation analysis using ARMS‐qPCR in relation to FNAC results and final histology after surgery

Cytology	Nondiagnostic (n = 12)		Benign (n = 38)		AUS/FLUS (n = 57)		FN/SFN (n = 5)		SUSP (n = 46)		Malignant (n = 129)		Total
Histology	BRAF^V600E^ (+)	BRAF^V600E^ (−)	BRAF^V600E^ (+)	BRAF^V600E^ (−)	BRAF^V600E^ (+)	BRAF^V600E^ (−)	BRAF^V600E^ (+)	BRAF^V600E^ (−)	BRAF^V600E^ (+)	BRAF^V600E^ (−)	BRAF^V600E^ (+)	BRAF^V600E^ (−)	
Malignant	3	2	8	6	25	6	1	3	33	6	115	14	222
Benign	0	7	2	22	1	25	0	1	1	6	0	0	65
Total (%)	3 (25)	9 (75)	10 (26)	28 (74)	26 (46)	31 (54)	1 (20)	4 (80)	34 (74)	12 (26)	115 (89)	14 (11)	287

Abbreviations: ARMS‐qPCR, amplification refractory mutation system‐quantitative real‐time polymerase chain reaction; AUS/FLUS, atypia of undetermined significance/follicular lesion of undetermined significance; FN/SFN, follicular neoplasm or suspicious for follicular neoplasm; FNAC, fine‐needle aspiration cytology; SUSP, suspicious for malignancy.

### 3.3 BRAF^V600E^ mutation analysis in FNAC specimens

The BRAF^V600E^ mutation rates were 25% (3/12) in the nondiagnostic cytology, 26% (10/38) in benign cytology, 46% (26/57) in AUS/FLUS cytology, 20% (1/5) in FN/SFN cytology, 74% (115/129) in SUSP cytology, and 89% in malignant cytology, respectively (Table 2).

### 3.4 BRAF^V600E^ mutation analysis in FFPE tissue samples

Among the 222 malignant nodules, 177 (79.7%) showed positive results for the BRAF^V600E^ mutation analysis in FFPE tissue samples. The prevalence of PTC in thyroid malignancy was 98.1% (218/222) and the positive mutation rate of BRAF^V600E^ in PTC was 81.2% (177 of 218). No positive BRAF^V600E^ mutation was found in the three FTCs, one ATC, and four nodular hyperplasias which showed BRAF^V600E^ mutation in FNAC specimens.

### 3.5 Consistency of BRAF^V600E^ mutation analysis between FNAC specimens and FFPE tissue samples

Consistency of BRAF^V600E^ mutation analysis between FNAC specimens and FFPE tissue samples occurred in 90.9% (261/287) of TNs while inconsistency in 9.1% (26/287) of nodules. The true‐positive mutation rate was 89.9% (170/189) and true‐negative mutation rate was 92.9% (91/98).

**Figure 2:**
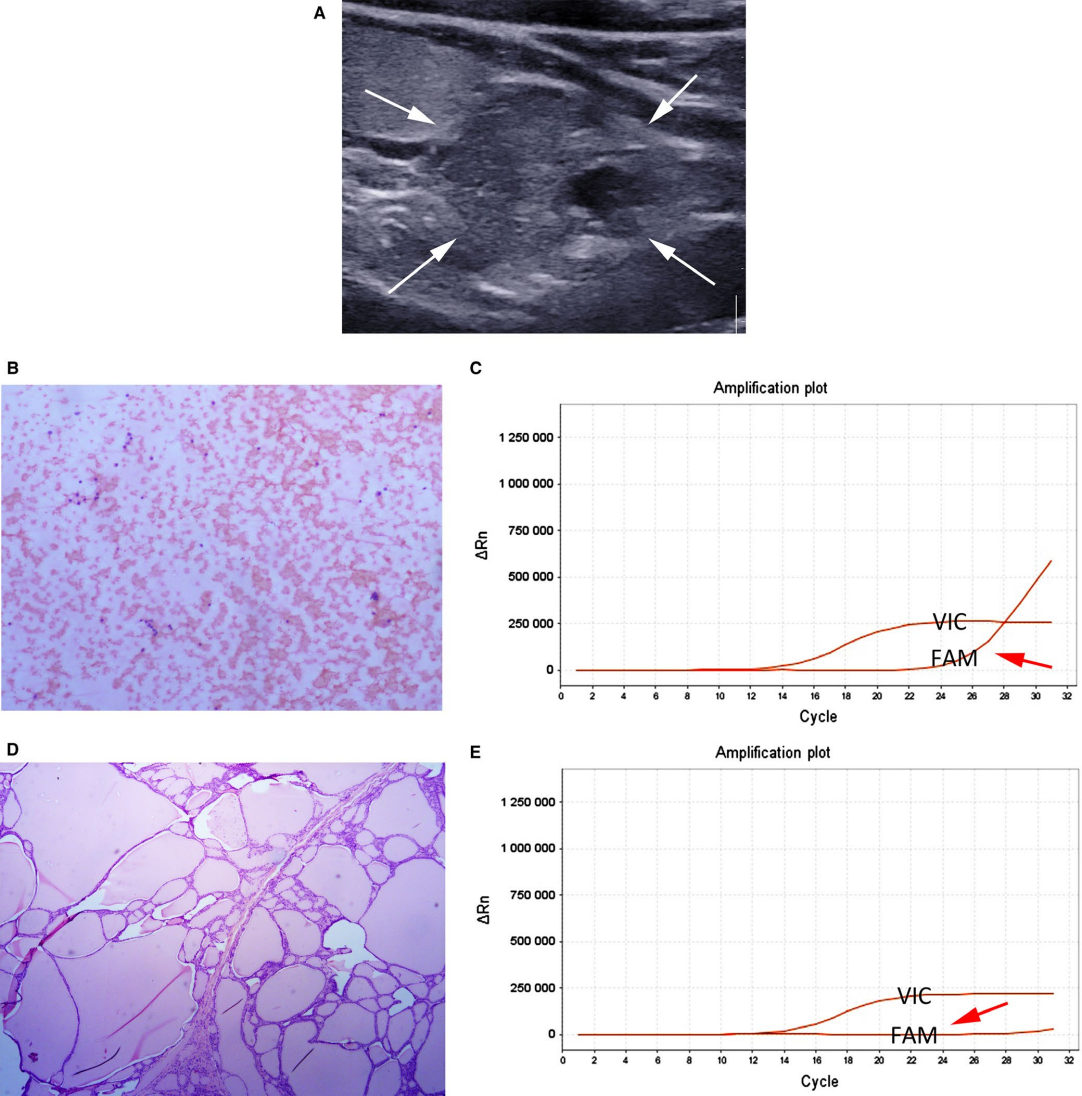
The false‐positive mutation for BRAF^V600E^ analysis using ARMS‐qPCR in preoperative FNAC specimen from a 41‐year‐old woman with nodular hyperplasia. A, Longitudinal US scan of left thyroid reveals a 26‐mm predominantly solid hypoechoic nodule (white arrow). B, The cytologic diagnosis from US‐guided FNAC is benign (H&E staining, ×100). C, BRAF^V600E^ analysis using ARMS‐qPCR in FNAC specimens shows positive mutation result (The orange amplification plot of the sample shows that the VIC signal and FAM signal [red arrow] rise, and the Ct value of the FAM signal is 25.53). D, Histology of surgical specimens shows nodular hyperplasia (H&E staining, ×50). E, BRAF^V600E^ analysis using ARMS‐qPCR in FFPE tissue samples shows negative mutation result (The orange amplification plot of the sample shows that the VIC signal rises but FAM signal [red arrow] does not rise)

**Figure 3:**
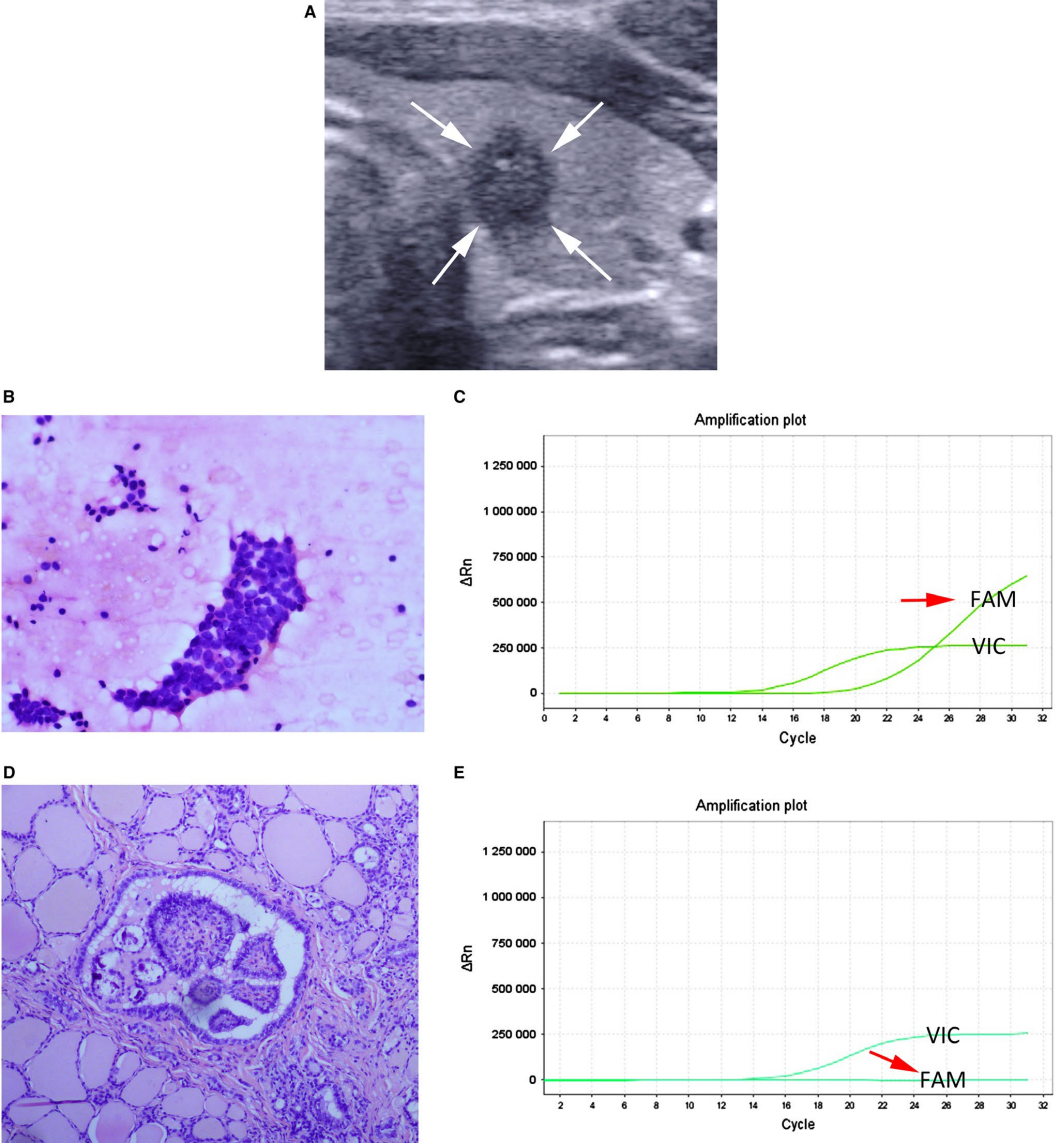
The false‐positive mutation for BRAF^V600E^ analysis using ARMS‐qPCR in preoperative FNAC specimen from a 28‐year‐old woman with classic PTC. A, Transverse US scan of left thyroid reveals a 6‐mm solid marked hypoechoic nodule (white arrow), with taller‐than‐wide shape and microcalcifications. B, The cytologic diagnosis from US‐guided FNAC is malignancy (H&E staining, ×400). C, BRAF^V600E^ analysis using ARMS‐qPCR in FNAC specimens shows positive mutation result (The light green amplification plot of the sample shows that the VIC signal and FAM signal [red arrow] rise, and the Ct value of the FAM signal is 21.75). D, Histology of surgical specimens shows classic PTC (H&E staining, ×100). E, BRAF^V600E^ analysis using ARMS‐qPCR in FFPE tissue samples shows negative mutation result (The light green amplification plot of the sample shows that the VIC signal rises but FAM signal [red arrow] does not rise)

The inconsistency group consisted of 26 nodules in 26 patients (24 women and 2 men; mean age ± standard deviation, 34 ± 14 years; range, 19‐75 years). In 73.1% (19/26) of the inconsistent nodules, BRAF^V600E^ mutations were detected in FNAC specimens, whereas not in FFPE tissue samples. These results were considered as false‐positive mutation. Thus, the false‐positive mutation rate was 10.1% (19/189). Of 19 false‐positive mutation nodules, four were nodular hyperplasias, one was minimally invasive FTC, and 14 were classic PTCs (Table 3) (Figures 2, 3).

**Table 3:** Characteristics of patients who showed false#x2010;positive mutation by ARMS‐qPCR for BRAF^V600E^ analysis in FNAC specimens

No.	Age (yr)	Sex	US size (mm)	FNAC results	BRAF^V600E^ mutation analysis		Histology
					FNAC Specimens ARMS‐qPCR	FFPE Tissue Samples ARMS‐qPCR	
1	53	F	12	II	+	−	Nodular Hyperplasia
2	41	F	26	II	+	−	Nodular Hyperplasia
3	50	F	6	III	+	−	Nodular Hyperplasia
4	32	F	5	V	+	−	Nodular Hyperplasia
5	25	F	5	VI	+	−	Classic PTC
6	75	F	6	V	+	−	Classic PTC
7	63	F	9	VI	+	−	Classic PTC
8	38	F	7	VI	+	−	Classic PTC
9	58	F	5	III	+	−	Classic PTC
10	28	F	6	VI	+	−	Classic PTC
11	33	F	5	VI	+	−	Classic PTC
12	29	F	6	VI	+	−	Classic PTC
13	42	F	9	VI	+	−	Classic PTC
14	35	F	8	II	+	−	Classic PTC
15	19	F	5	V	+	−	Classic PTC
16	56	F	5	VI	+	−	Classic PTC
17	46	F	9	VI	+	−	Classic PTC
18	57	F	10	VI	+	−	Classic PTC
19	23	M	15	IV	+	−	Minimally Invasive FTC

Abbreviations: ARMS‐qPCR, amplification refractory mutation system‐quantitative real‐time polymerase chain reaction; FNAC, fine‐needle aspiration cytology; FTC, follicular thyroid carcinomas; PTC, papillary thyroid carcinoma; US, ultrasound.

**Figure 4:**
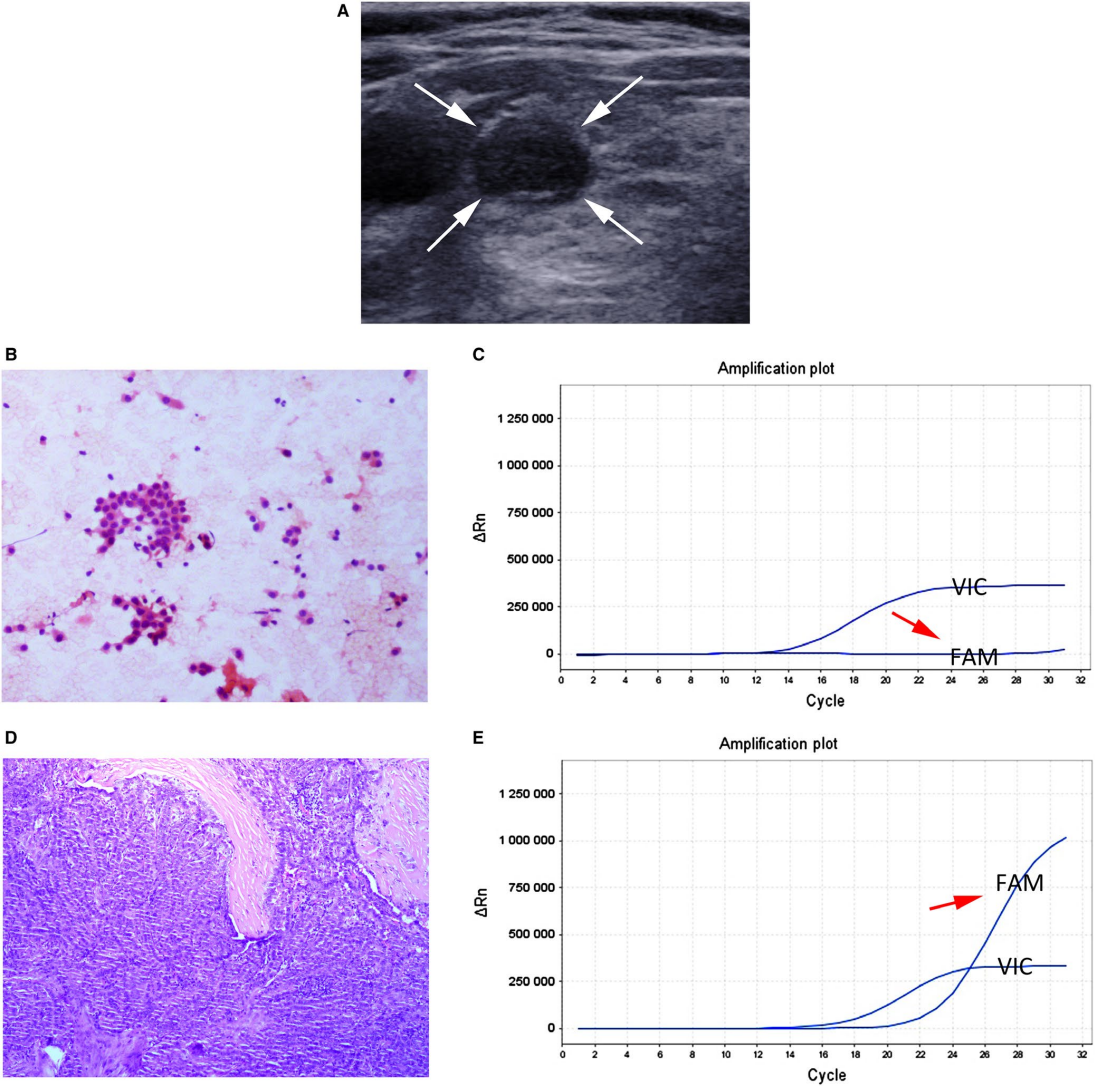
The false‐negative mutation for BRAF^V600E^ analysis using ARMS‐qPCR in preoperative FNAC specimen from a 54‐year‐old woman with classic PTC. A, Transverse US scan of right thyroid reveals a 9‐mm solid marked hypoechoic nodule (white arrow) in the inhomogeneous thyroid background. B, The cytologic diagnosis from US‐guided FNAC is malignancy (H&E staining, ×100). C, BRAF^V600E^ analysis using ARMS‐qPCR in FNAC specimens shows negative mutation result (The purple amplification plot of the sample shows that the VIC signal rises but FAM signal [red arrow] does not rise). D, Histology of surgical specimens shows classic PTC (H&E staining, ×100). E. BRAF^V600E^ analysis using ARMS‐qPCR in FFPE tissue samples shows positive mutation result (The purple amplification plot of the sample shows that the VIC signal and FAM signal [red arrow] rise, and the Ct value of the FAM signal is 22.21)

In the remaining 26.9% (7/26) nodules, BRAF^V600E^ mutation was negative in FNAC specimens, whereas positive in FFPE tissue samples, indicating false‐negative mutation. Thus, the false‐negative mutation rate was 7.1% (7/98). Of the seven false‐negative mutation nodules, six were classic PTCs and one was mixture of follicular variant of PTC and classic PTC (Table 4) (Figure 4).

**Table 4:** Characteristics of patients who showed false‐negative mutation by ARMS‐qPCR for BRAF^V600E^ analysis in FNAC specimens

No.	Age (yr)	Sex	US size (mm)	FNAC results	BRAF^V600E^ mutation analysis		Histology
					FNAC specimens ARMS‐qPCR	FFPE Tissue Samples ARMS‐qPCR	
1	30	F	5	VI	−	+	Classic PTC
2	36	F	10	V	−	+	Classic PTC
3	54	M	5	III	−	+	Classic PTC
4	54	F	9	VI	−	+	Classic PTC
5	37	F	5	II	−	+	Classic PTC
6	54	F	9	V	−	+	Classic PTC
7	54	F	18	V	−	+	Mixture of FVPTC and Classic PTC

Abbreviations: ARMS‐qPCR, amplification refractory mutation system‐quantitative real‐time polymerase chain reaction; FFPE, formalin‐fixed, paraffin‐embedded; FNAC, fine‐needle aspiration cytology; PTC, papillary thyroid carcinoma; FVPTC, follicular variant of PTC; US, ultrasound.

### 3.6 Diagnostic performances of FNAC, BRAF^V600E^ analysis in FNAC specimens, BRAF^V600E^ analysis in FFPE tissue samples, and combination of FNAC and BRAF^V600E^ analysis

Concerning the diagnostic performance for TNs, FNAC showed a sensitivity of 75.7%, specificity of 89.2%, PPV of 96%, NPV of 51.7%, and accuracy of 78.7% in diagnosing thyroid malignancy, with the histopathological analysis after surgery as the reference standard (Table 5).

**Table 5:** Diagnostic performances of FNAC, BRAF^V600E^ analysis in FNAC specimens, BRAF^V600E^ analysis in FFPE tissue samples, and the combination of FNAC and BRAF^V600E^ analysis for predicting thyroid malignancy

Diagnostic modality	Sensitivity	Specificity	PPV	NPV	Accuracy
FNAC	75.7% (168/222)	89.2% (58/65)	96% (168/175)	51.7% (58/112)	78.7% (226/287)
BRAF^V600E^ analysis in FNAC specimens	83.3% (185/222)	93.8% (61/65)	97.9% (185/189)	62.2% (61/98)	85.7% (246/287)
FNAC + BRAF^V600E^ analysis in FNAC specimens	92.3% (205/222)	84.6% (55/65)	95.3% (205/215)	76.4% (55/72)	90.6% (260/287)
BRAF^V600E^ analysis in FFPE tissue samples	79.3% (176/222)	100% (65/65)	100% (176/176)	58.6% (65/111)	84.0% (241/287)
FNAC + BRAF^V600E^ analysis in FFPE tissue samples	91.9% (204/222)	89.2% (58/65)	96.7% (204/211)	76.3% (58/76)	91.3% (262/287)

Abbreviations: FFPE, formalin‐fixed, paraffin‐embedded; FNAC, fine‐needle aspiration cytology; NPV, negative predictive value; PPV, positive predictive value.

Adding the molecular test of BRAF^V600E^ mutation analysis to FNAC significantly improved the diagnostic performance, with sensitivity increasing from 75.7% to 92.3% (*P* < .001), and accuracy from 78.7% to 90.6% (*P* < .001) (Table 5). Forty nodules with negative cytology (nondiagnostic, benign, AUS/FLUS, and FN/SFN) presented positive results for BRAF^V600E^ by ARMS‐qPCR analysis. These patients underwent thyroidectomy. Three showed benign (all nodular hyperplasias) and 37 showed malignancy in the final histology (three false‐positive mutation cases including one minimally invasive FTC and two classic PTCs).

To evaluate whether the inconsistency of BRAF^V600E^ mutation analysis before and after surgery would affect the diagnostic performance of combination of FNAC and BRAF^V600E^ mutation analysis, we performed the following comparison study and found that no significant differences were found between the combination of FNAC and BRAF^V600E^ mutation analysis in FNAC specimens and the combination of FNAC and BRAF^V600E^ mutation analysis in FFPE tissue samples (sensitivity: 92.3% vs 91.9%; accuracy: 90.6% vs 91.3%; both *P* > .05) (Table 5).

## 4 DISCUSSION

In the present study, 98.1% of thyroid cancers were PTCs and the prevalence of BRAF^V600E^ in PTC patients was 81.2%, which were consistent with other reports from China.^20^, ^21^, ^22^, ^25^ Similarly, 95% or more of thyroid cancers are PTCs and 80% or more of PTCs harboring the BRAF^V600E^ mutation in another Eastern country of South Korea.^6^, ^17^ The prevalence of PTC in thyroid cancer (80%‐90%) and the prevalence of BRAF^V600E^ mutation in PTC (30%‐50%) in Western countries, however, are much lower than that in Eastern Asian countries.^12^, ^17^ These differences may originate from the detection methods used and the variable prevalence of genetic alterations in thyroid malignancy according to ethnicity, geographic area, and iodine consumption.

Comparing with previous studies,^5^, ^6^, ^12^, ^17^ we found a higher false‐positive mutation rate using ARMS‐qPCR (10.1%) than other PCR‐based methods (0.08%‐5.4%). To the best of our knowledge, those studies evaluated BRAF^V600E^ mutation only in FNAC specimens, but not in both FNAC specimens and surgical specimens. Surgically proven benign cases of false‐positive BRAF^V600E^ mutation have been documented in the literature. Using preoperative conventional Sanger sequencing for BRAF^V600E^ mutation testing, Chung et al 17 found one false‐positive mutation case with indeterminate FNAC result, which was confirmed to be an atypical hyperplastic nodule in the background of Hashimoto's thyroiditis. Kim et al 26 reported two false‐positive mutation cases among 17 nodules treated by thyroidectomy, which were confirmed to be an adenomatous hyperplasia with underlying lymphocytic thyroiditis and a fibrotic nodule with dense calcification. Using the preoperative DPO‐based multiplex PCR analysis, Direnzo et al 27 reported one false‐positive mutation case that was proven to be an adenomatoid nodule with indeterminate FNAC result. Kim et al 5 reported five‐ false positive mutation cases that surgery revealed to be benign (one follicular adenoma and four nodular hyperplasias). In our study, we also observed four false‐positive mutation cases, which were confirmed to be benign (all nodular hyperplasias). The false‐positive mutation results for benign nodules may cause unnecessary thyroid surgery. In additional, we found 15 false‐positive mutation cases, which were confirmed to be malignant but no BRAF^V600E^ mutations were detected in FFPE tissue samples (one minimally invasive FTC and 14 classic PTCs). Thus, the real false‐positive mutation rate of BRAF^V600E^ testing methods in FNAC specimens may be underestimated. Additionally, this might be attributed to the difference in detection mechanisms of the difference assays. The ARMS‐qPCR, similar to DPO‐based multiplex PCR, is based on the ARMS method which is known to have a relatively high false‐positive rate. The false‐positive mutation is a result of the overly sensitive assay. On the other hand, it is noteworthy that of the 19 false‐positive mutation nodules, 15 (79%, 15/19) were malignant, including one minimally invasive FTC and 14 classic PTCs. Therefore, the false‐positive mutation results seem not affect the following management strategy.

Seven false‐negative mutation cases were found, which were confirmed to be malignant and BRAF^V600E^ mutations were detected in FFPE tissue samples (six were classic PTCs and one was a mixture of follicular variant of PTC and classic PTC). One of the possible reasons might be that the FNAC specimen was admixed with normal thyroid cells, other type cells, and blood. And, the LOD for mutant alleles was as low as 1% for the ARMS‐qPCR method used in the present study.^28^, ^29^ Thus, to reduce the possibility of false‐negative results due to low numbers of tumor cells, it is necessary to select more regions of representative slides for assuring adequate lesional thyrocytes in “manual macrodissection” method.

The added value of BRAF^V600E^ test is its ability to detect PTCs that might have been missed by FNAC. Examples may include tumors with indeterminate (AUS/FLUS and FN/SFN), benign, and nondiagnostic cytology. In our study, the molecular test increased the sensitivity of FNAC from 75.7% to 92.3%. Diagnostic accuracy also increased from 78.7% to 90.6%. These findings were consistent with those of Kim et al,^5^ who reported gains in sensitivity (from 67.5% to 89.6%) and accuracy (from 90.9% to 96.6%). The greatest increase in sensitivity came from the detection of PTC in the patients with indeterminate (AUS/FLUS and FN/SFN) cytology of FNAC results. About 10%‐75% nodules with indeterminate (AUS/FLUS and FN/SFN) cytology are follicular neoplasms which cannot be reliably differentiated from malignancy by FNAC and intraoperative frozen section analysis.^30^ This creates a difficult clinical dilemma concerning the extent of surgery (total thyroidectomy or hemithyroidectomy). The molecular test for indeterminate FNAC result patients may be beneficial in planning the extent of surgery.

It is interesting that no significant difference in sensitivity and accuracy were found between the combination of FNAC and the BRAF^V600E^ mutation analysis in FNAC specimens and the combination of FNAC and the BRAF^V600E^ mutation analysis in FFPE tissue samples. The reason is that the majority of (17/26) inconsistent cases show malignant or suspicious malignant cytology in FNAC results, in which molecular test did not change the cytological judgment for TNs. These results suggested that those false‐positive and false‐negative mutation cases using ARMS‐qPCR for the BRAF^V600E^ analysis in preoperative FNAC specimen would not affect the main outcomes of molecular test as a complementary diagnostic tool to FNAC in the diagnosis of TNs. Meanwhile, considering the final assessment of TNs, the value of molecular test should be evaluated in combination with FNAC result.

At last, several limitations are existed in this study. First, surgery‐proven benign TNs were not tested for BRAF^V600E^ mutation analysis except for those showing BRAF^V600E^ mutation in FNAC specimens. As we know, no nodules reported as benign tested positive for the BRAF^V600E^ mutation. Second, allelic fraction was able to be analyzed by conventional Sanger sequencing, whereas not applicable for ARMS‐qPCR, and the comparison between ARMS‐qPCR and other molecular techniques, such as ddPCR which is a novel and ultrasensitive method for BRAF^V600E^ mutation analysis in FNAC specimens 19, was not carried out in the current study. Thus, it should be evaluated in further studies. Third, the “gold standard” for determining detection accuracy of ARMS‐qPCR for BRAF^V600E^ mutation analysis from FNAC specimens is ARMS‐qPCR from FFPE specimen in this study. Conventional Sanger sequencing is a highly reliable and widely used method to detect the BRAF^V600E^ mutations in FFPE specimen,^6^, ^14^ which might be used as the “gold standard” in this study to explicitly reflect detection accuracy of ARMS‐qPCR for BRAF^V600E^ mutation analysis from FNAC specimens. In addition, this work was a single institution study. Therefore, a larger multicenter study for evaluation the false‐positive and false‐negative mutation rates of the BRAF^V600E^ analysis using ARMS‐qPCR in preoperative FNAC specimen is mandatory in the near future.

In conclusion, FNAC combined with preoperative BRAF^V600E^ mutation analysis can significantly increase the diagnostic performance in comparison with FNAC alone. False‐positive and false‐negative BRAF^V600E^ mutation results are present in FNAC specimens using ARMS‐qPCR, whereas it does not affect the added diagnostic value of BRAF^V600E^ mutation analysis to FNAC for evaluation of TNs.

## CONFLICT OF INTEREST

The authors do not have any conflict of interest to declare.

## AUTHOR CONTRIBUTIONS

Concept and design: H‐X X. BRAF^V600E^ mutation analysis: R‐YX. Collection and assemble of data: C‐KZ, J‐YZ, and QW. Data analysis: C‐KZ, J‐YZ, and L‐PS. Manuscript written: C‐KZ. Reviewed the manuscript: All authors.

## Data Availability

The data are available from the corresponding author when upon reasonable requests.
